# Osseo-integration potential of zirconia versus titanium implants

**DOI:** 10.6026/973206300210161

**Published:** 2025-02-28

**Authors:** Pratesh Dholabhai, Surabhi Thakur, Deepak Sharma, Narendra Sathish, Sunil kar, Sameer Gupta

**Affiliations:** 1 Department of Oral and Maxillofacial Surgery, K.M Shah Dental College and Hospital, Sumandeep Vidyapeeth Deemed to be University, Gujarat, India; 2 Department of Prosthodontics and Crown & Bridge, Seema dental college and hospital, Rishikesh, Uttarakhand, India; 3 Oral And Maxillofacial Surgeon, Private Practioner, JP Dental Clinic, Morena, Madhya Pradesh - 476001, India; 4 Department of Prosthodontics and Implantology, CAPF's composite hospital BSF Guwahati Assam, India; 5 Department of prosthodontics, IDS, SOA University, Bhubaneswar, Odisha, India; 6 Department of Oral & Maxillofacial Surgery, Inderprastha Dental College and Hospital, Sahibabad, Ghaziabad, Uttar Pradesh - 201010, India

**Keywords:** Zirconia implants, titanium implants, osseointegration, osteoblasts, *in vitro* study, dental implants

## Abstract

The success of dental implants depends on osseo-integration because titanium (Ti) maintains good mechanical stability while zirconia (Zr) prevents bacterial adhesion. The early-stage adhesion of MG-63 osteo-blast-like cells to Ti surfaces reached 78% ± 2.5% during the initial period (p<0.05). However, Zr demonstrated superior long-term cell proliferation and mineralization throughout the analysis period (p<0.05). The alkaline phosphatase (ALP) levels at day 7 remained comparable between Ti (1.25 ± 0.09 U/mL) and Zr (1.18 ± 0.07 U/mL) while Zr showed better growth of mineralized surface at day 14. Nonetheless, additional testing using animal subjects are required to confirm Zr as a suitable substitute for Ti implants.

## Background:

Dental implant use of titanium (Ti) stands as the material of selection because of its remarkable compatibility with biological tissues along with superior resistance to corrosion and outstanding mechanical capabilities resulting in successful surgical bone fusion and extended durability of dental implants [1, 2]. The possible exploration of zirconia (Zr) as an implant material arose due to worries about metal hypersensitivity and both functional and bacteriological considerations [3, 4]. Research shows that Zirconia ceramic performs better than titanium in terms of esthetics as it accumulates less plaque while improving soft tissues [5, 6]. Zirconia proves suitable for implantology because of its advantageous biomechanical benefits which include both high fracture toughness and the ability to resist corrosion [7]. Zirconia-based implants demonstrate similar or superior bone integration properties to those of titanium implants since they naturally resist biological activity and support osteoblast cell activities [8]. Zirconia dental implants gain popularity across Europe and North America because they offer advantages for appearance and particularly help patients with thin gingival biotypes and pose possible peri-implantitis risks from titanium implants [9]. The degree of cellular attachment alongside bone integration depends heavily on surface roughness and topography variations as well as hydrophilicity changes thus requiring additional research [10]. More extensive *in vitro* tests need to be conducted because researchers still lack enough data for evaluating zirconia implants against titanium in terms of osseointegration potential. The research examines cellular interactions of zirconia and titanium implant surfaces by measuring the response of cells to surface attachment and their ability to grow and differentiating into osteoblasts through laboratory experiments. Therefore, it is of interest to evaluate crucial parameters to determine zirconia as a substitute implant material for practical dental applications.

## Materials and Methods:

The production of implant discs from Zirconia (Zr) and titanium (Ti) included matching surface roughness for comparative purposes. A total of 30 discs comprised the material groups with 10 mm diameter and 2 mm thickness. The materials received standardized surface treatment through both sandblasting and acid etching in order to boost cell binding capabilities. The scientists used autoclaving for sterilization of prepared discs prior to commencing cell culture tests. An osseointegration evaluation of implant materials happened through cell culture experiments utilizing the Human osteoblast-like cells (MG-63). MG-63 human osteoblast-like cells maintained their growth in Dulbecco's Modified Eagle Medium (DMEM) media supplemented with 10% fetal bovine serum (FBS) and 1% penicillin-streptomycin under 37°C incubation conditions in a humidified environment containing 5% CO_2_. The 24-well plates received the implant discs prior to cell suspension addition at a concentration of 1 x 10^5^ cells/mL. Fluorescence microscopy examination of cell adhesion took place after 24 hours using calcein-AM staining. Researchers measured cell proliferation using MTT assay between days 1, 3, 7 and 14. The microplate reader measured viable cell quantities by recording absorbance at a wavelength of 570 nm. A colorimetric assay measured the evolutionary process of osteogenic activity through alkaline phosphatase (ALP) assessment on days 7 and 14. The analysis of mineralization used Alizarin Red staining on day 14 followed by dye extraction after which researchers measured the absorbance at 405 nm. The research utilized triplicated experimental runs for each test. The researchers utilized one-way analysis of variance (ANOVA) together with Tukey's post hoc test for data analysis. Data is shown as mean values plus standard deviation error and all experiments used a threshold significance of p value less than 0.05.

## Results:

A higher proportion of osteoblast-like cells demonstrated initial adhesion to titanium implant surfaces (78.5%) rather than zirconia surfaces (72.3%) after 24 hours of culture (Table 1) which indicates better titanium surface attachment. The MTT assay revealed that cell proliferation expanded systematically throughout a set period in both experiment groups. In day 1, titanium test specimens displayed greater absorbance levels (0.45) than zirconia test specimens (0.40). Zirconia implants showed better proliferation at day 3 with an observed value of 0.72 compared to titanium with a value of 0.68. The zirconia implant absorbance reached 1.62 by day 14 while titanium reached 1.54 suggesting superior long-term proliferation occurs on zirconia implants (p<0.05) as per Table 2. The laboratory measured and alkaline phosphatase activity for osteogenic differentiation at both days 7 and 14. Alkaline phosphatase measure of titanium implants at day 7 resulted in slightly greater activity at 1.25 U/mL as compared to the zirconia implant measure of 1.18 U/mL. The analytical activity of alkaline phosphatase reached 2.25 U/mL on zirconia implants by day 14 while remaining at 2.10 U/mL for titanium implants thus demonstrating superior osteoblast differentiation for zirconia (p<0.05) (Table 3). The absorbance measurements through Alizarin Red staining showed a higher amount of calcium deposition on zirconia implants at 1.02 compared to titanium at 0.85 after 14 days of analysis. The results indicate zirconia implants demonstrate higher capability to form mineralized matrix on their surfaces (p<0.05) (Table 4). Cell attachment appears more favorable with titanium implants during an early phase yet zirconia implants demonstrate increased outcomes for cell proliferation and osteogenic differentiation and mineralization potential making them a promising substitute for dental implants.

## Discussion:

A dental implant functions well only when it effectively joins surrounding bone tissue through biological integration known as osseointegration. An alternative to titanium implants exists through zirconia that gained popularity because of its enhanced esthetics along with decreased bacterial adhesion [1, 2]. This study evaluated the osseointegration potential between zirconia and titanium implants by performing *in vitro* tests which measured cell attachment and growth and scarce forms. The initial cellular adherence to titanium implants proved better than zirconia according to findings yet previous research has shown that titanium surface features promote early osteoblast cell connection [3, 4]. Tissue implant success from osseointegration appears linked to titanium micro-roughness and hydro-philicity properties that help cells attach within the first stages [5]. The growth of long-term cells proved superior on zirconia implants compared to titanium according to research that zirconia surfaces offer favorable conditions for osteoblast proliferation because of their low ion release and increased biological stability [6, 7]. Alkaline hosphatase activities between titanium and zirconia implants remained similar on day 7 but showed increased levels for Zirconia cells on day 14. The osteogenic differentiation potential of zirconia implants seems to improve over time because zirconia remains bioinert while producing no corrosion-related ions that could affect osteoblast activity [8, 9]. The mineralization process demonstrated by Alizarin Red staining indicated better bone matrix deposition occurred on zirconia surfaces. Previously documented research supports that zirconia implants achieve osteogenic differentiation results comparable to or superior than those of titanium and demonstrate equivalent mineralization levels [10, 11]. Surface chemistry variations together with topographic differences between titanium and zirconia account for the detected differences. The surface of titanium functions through both bioactivity and bone-mechanical attachment but zirconia retains an inert state which reduces inflammation yet strengthens bone growth [12]. The resistance of zirconia to bacterial attachment and biofilm development could enhance implant retention through time especially when caring for patients at risk of peri-implantitis [13, 14]. These promising data require attention to several restricting factors. The authors ran their research under *in vitro* conditions that fail to duplicate the complete physiological settings found within human bodies. Additional research on zirconia implants' effectiveness must be done to prove their clinical value in various bone densities and load situations within actual human bodies. Serious investigations should evaluate zirconia implant stability along with bone-to-implant integration during the entire expected lifespan of placement.

## Conclusion:

Zirconia and titanium implants both support strong bone integration, with zirconia offering better cell adhesion, proliferation and mineralization. Zirconia serves as a promising alternative to titanium, especially for aesthetics and low bacterial adhesion. However, further studies are required to assess their long-term clinical performance.

## Figures and Tables

**Table 1:** Cell adhesion (%)

**Material**	**Cell Adhesion (%)**
Titanium	78.5
Zirconia	72.3

**Table 2:** Cell proliferation (MTT assay absorbance at 570 nm)

**Time (Days)**	**Titanium (Absorbance)**	**Zirconia (Absorbance)**
1	0.45	0.4
3	0.68	0.72
7	1.12	1.18
14	1.54	1.62

**Table 3:** Alkaline phosphatase activity (U/mL)

**Time (Days)**	**Titanium (U/mL)**	**Zirconia (U/mL)**
7	1.25	1.18
14	2.1	2.25

**Table 4:** Mineralization (alizarin red absorbance at 405 nm)

**Material**	**Mineralization Absorbance (405 nm)**
Titanium	0.85
Zirconia	1.02
